# Optimizing Preclinical Skill Assessment for Handpiece-Naïve Students: A Strategic Approach

**DOI:** 10.3390/dj13080363

**Published:** 2025-08-11

**Authors:** Reinhard Chun Wang Chau, Szabolcs Felszeghy, Maria F. Sittoni-Pino, Santiago Arias-Herrera, Sompop Bencharit, Margrit Maggio, Murat Mutluay, David P. Rice, Walter Yu Hang Lam, Sıla Nur Usta, Barry F. Quinn, Jorge Tricio, Masako Nagasawa, Mihaela Pantea, Marina Imre, Ana Maria Cristina Tancu, Amitha Ranauta, Arzu Tezvergil-Mutluay, Satu Korpisaari, Kaisa Leinonen, Mikko Liukkonen, Outi S. Huhtela, Ulf T. Örtengren, Peter Lingström

**Affiliations:** 1 Faculty of Dentistry, University of Hong Kong, Hong Kong SAR 999077, China; rcwchau@hku.hk (R.C.W.C.); retlaw@hku.hk (W.Y.H.L.); 2 Institute of Dentistry, School of Medicine, University of Eastern Finland, 70211 Kuopio, Finland; satu.korpisaari@uef.fi (S.K.); kaisa.leinonen@uef.fi (K.L.); outi.huhtela@uef.fi (O.S.H.); 3 Department of Dentistry, Faculty of Health Sciences, Universidad Europea de Valencia, 46010 Valencia, Spain; mariaflorencia.sittoni@universidadeuropea.es (M.F.S.-P.); santiagoemilio.arias@universidadeuropea.es (S.A.-H.); 4 Workman School of Dental Medicine, High Point University, High Point, NC 27262, USA; sbenchar@highpoint.edu (S.B.); mmaggio@highpoint.edu (M.M.); 5 Helsinki University Hospital, University of Helsinki, 00280 Helsinki, Finland; murat.mutluay@helsinki.fi (M.M.); david.rice@helsinki.fi (D.P.R.); 6 Gulhane Faculty of Dentistry, University of Health Sciences, Ankara 06018, Turkey; silanur.usta@sbu.edu.tr; 7 School of Dentistry, University of Liverpool, Liverpool L7 8YA, UK; barry.quinn@liverpool.ac.uk; 8 Faculty of Health and Dentistry, Universidad de los Andes, Santiago 7620001, Chile; jtricio@uandes.cl; 9 Division of Bio-Prosthodontics, Faculty of Dentistry, Graduate School of Medical and Dental Sciences, Niigata University, Niigata 951-8514, Japan; nagasawa@dent.niigata-u.ac.jp; 10 Faculty of Dentistry, “Carol Davila” University of Medicine and Pharmacy, 020021 Bucharest, Romania; mihaela.pantea@umfcd.ro (M.P.); marina.imre@umfcd.ro (M.I.); anamaria.tancu@umfcd.ro (A.M.C.T.); 11 Institute of Dentistry, Queen Mary University of London, London E1 4NS, UK; a.ranauta@qmul.ac.uk; 12 Institute of Dentistry, University of Turku, 20500 Turku, Finland; arztez@utu.fi; 13 Institute of Clinical Medicine, School of Medicine, University of Eastern Finland, 70211 Kuopio, Finland; mikko.liukkonen@uef.fi; 14 Department of Cariology, Institute of Odontology, Sahlgrenska Academy, University of Gothenburg, 40530 Gothenburg, Sweden; ulf.ortengren@mau.se (U.T.Ö.); peter.lingstrom@odontologi.gu.se (P.L.); 15 Department of Material Science and Technology, Faculty of Odontology, Malmö University, 20506 Malmö, Sweden

**Keywords:** dental education, haptics, preclinical training, manual skills

## Abstract

**Background**: Preclinical dental training requires simulation-based tools to develop fine motor skills, but traditional models like plastic teeth often lack realistic tactile feedback, and systematic evaluations of multi-layered drilling plates are scarce. This study aimed to evaluate the educational utility and perceived realism of a novel multi-layered drilling plate designed to simulate enamel, dentin, and pulp, with null hypotheses that it would not differ in realism from natural dental tissues or in educational utility from existing tools. **Methods**: Seventy dental educators (mean preclinical teaching experience: 112.9 ± 116.7 months) from 14 institutions across four continents assessed the plates using standardized protocols. Statistical analysis (Mann–Whitney U Test) was performed to analyze the results. **Results**: Quantitative ratings (1–10 scale) showed high mean scores for drilling quality (enamel: 7.80 ± 1.55, dentin: 7.27 ± 1.94, pulp: 7.48 ± 2.33), surface smoothness (enamel: 8.17 ± 1.55, dentin: 8.17 ± 1.57), and ergonomic visibility (8.56 ± 1.58), with 90% passing grades, rejecting the null hypothesis of no difference in educational utility. Tissue transition scores (enamel/dentin: 7.09 ± 2.56; dentin/pulp: 6.86 ± 2.46) showed significant differences (*p* < 0.05) in realism from natural tissues, rejecting the null hypothesis of no difference. Inter-rater reliability was poor (Krippendorff’s alpha: 0.449 for failing scores, 0.211 for passing scores). Qualitative feedback praised ease of use but noted limitations in dentin haptic simulation. **Conclusions**: The drilling plate shows promise for skill development, though without controlled comparisons to existing tools, its relative efficacy remains preliminary. Further research on student outcomes and tool refinement is needed to validate its use in dental education.

## 1. Introduction

The development of fine motor skills is a cornerstone of dental education, enabling students to perform precise clinical procedures that require discerning the positions, sizes, shapes, and tactile properties of dental tissues [1,2]. Traditional preclinical training relies on phantom head simulators and plastic teeth, which often fail to replicate the tactile feedback of natural dental tissues, particularly the distinct hardness of enamel, dentin, and pulp [3,4]. Advances in dental technology have introduced virtual reality simulators with haptic interfaces (VR-haptics), which enhance skill acquisition but cannot fully replace physical simulation [5,6]. These limitations can impede students’ ability to develop the tactile sensitivity needed for procedures like caries removal, which demand differentiation between healthy and carious tissues [7,8,9].

To address these challenges, multi-layered drilling plates have been developed to mimic the mechanical properties of dental hard tissues, incorporating distinct layers for enamel, dentin, and pulp, though replicating soft tissues remains a challenge [10,11]. Early in dental education, students practice preparing geometric shapes in drilling plates inserted into phantom heads, allowing handpiece-naïve learners to hone skills in a controlled environment [12,13]. Early during practical education, the students also practice the preparation of geometric shapes in drilling plates on a bench and not only inserted into phantom heads [13]. These drilling plates, designed with varying hardness levels to simulate clinical conditions, provide a foundational step for preclinical training, bridging the gap between theoretical knowledge and clinical application, yet systematic evaluations of their efficacy and realism are limited [14,15].

The evolving landscape of dental education emphasizes the integration of traditional hands-on methods with innovative technologies, including virtual reality (VR), haptic simulators, and AI-powered platforms [16]. For example, AI-designed dental crowns and the resulting virtual patient models may enhance dental training by providing realistic, patient-specific scenarios that can improve both clinical precision and decision-making skills [17]. This blended approach not only enhances technical proficiency, but also supports student well-being by incorporating stress-reduction strategies, such as background music and mindfulness techniques, which have been shown to accelerate skill acquisition and reduce anxiety [18]. While digital tools offer individualized feedback and low-risk practice environments, traditional mentorship remains essential for fostering clinical judgment and professional values [19,20]. This synergy creates a comprehensive educational framework that prepares students for clinical practice.

To date, there is a lack of nationally or internationally available data specifically evaluating the use of drilling plates in preclinical training. While various preclinical training tools are used nationally and internationally, systematic studies comparing their efficacy, cost, durability, or simulation realism are scarce. This gap highlights the need for further research to establish standardized metrics and comparative analyses, as addressed in the present study. This study evaluated the educational utility and perceived realism of a novel multi-layered drilling plate designed to simulate the tactile and structural properties of human dental hard tissues. Unlike conventional plastic teeth, which lacked adequate differentiation between enamel, dentin, and pulp, this drilling plate incorporated three distinct layers to enhance preclinical training. The null hypotheses of this study were that the novel multi-layered drilling plate would not differ in perceived realism from natural dental tissues and that it would not differ in educational utility from existing preclinical training tools, as assessed by dental educators. Through a multi-institutional investigation involving dental educators from diverse pedagogical and cultural backgrounds, this study assessed the generalizability, realism, and efficacy of the drilling plate as a training tool, aiming to advance the integration of innovative and traditional methods in dental education.

## 2. Methods

### 2.1. Study Design and Ethical Considerations

This multi-institutional study was designed based on established guidelines for educational research, ensuring methodological rigor, reproducibility, and ethical compliance. All procedures adhered to the principles outlined in the Declaration of Helsinki [21]. Informed consent was obtained from all participants, and the study protocol was approved by the Institutional Review Board of the University of Eastern Finland (Reference Number: 40/2023).

### 2.2. Participant Recruitment

All 30 dental institutions of the Digital, VR, and Haptic Thinkers Global Network were invited to participate in this study. The inclusion criteria for individual participants were as follows [22]:

1. Those who hold a primary dental degree;
2. Those who are involved in preclinical teaching.

The exclusion criteria were as follows:

1. Those who have not received a recognized primary dental qualification;
2. Those who have no experience in preclinical teaching.

From those expressing interest, 15 dental institutions were randomly selected using a randomization table [23], from four different continents and representing a broad range of pedagogical perspectives.

### 2.3. Standardization and Pre-Study Calibration

**Figure A1:**
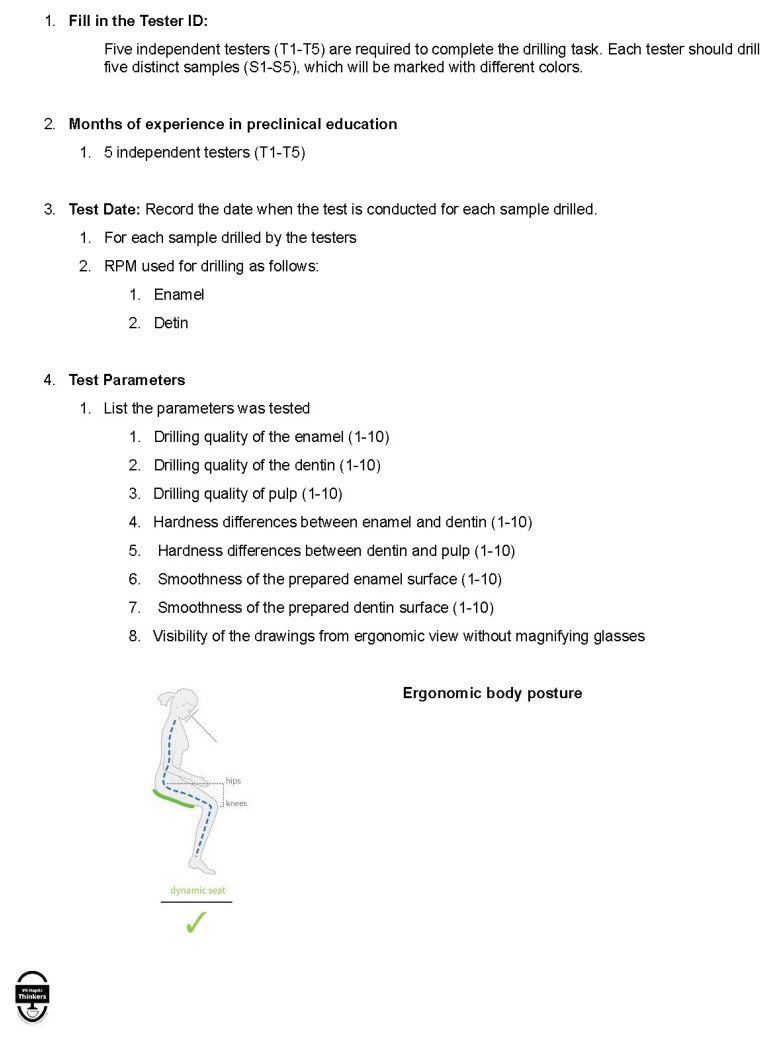
The written guideline with standard operating procedures used in the study.

**Figure A2:**
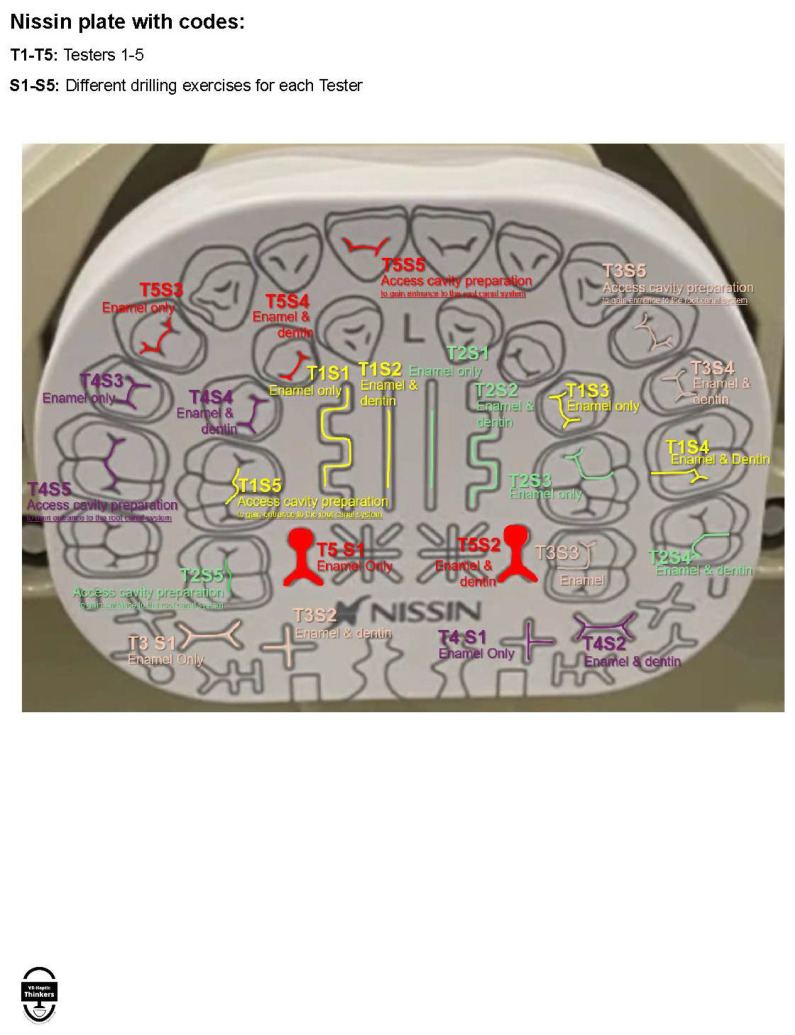
The colored drilling guideline used in the study.

To ensure uniform execution, a preparatory video meeting was conducted via Microsoft Teams, where procedural steps were thoroughly reviewed and calibrated. A detailed written guideline, including standard operating procedures and the assessor form (Figure A1), was distributed to all participants. The guideline specified that five independent testers (T1–T5) at each institution would evaluate five distinct drilling samples (S1–S5), each marked with a different color for identification (Figure A2). All participants were instructed to complete each sampling procedure within 2 minutes. This calibration process minimized inter-operator variability and ensured adherence to EU standards for testing dental materials.

### 2.4. Materials and Equipment

**Figure 1:**
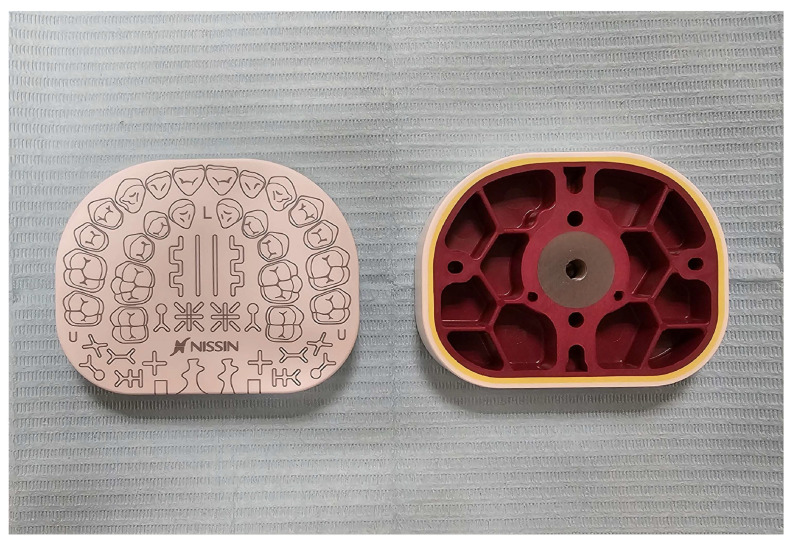
Front and rear views of the drilling plates assessed in the study. Dimensions: 16 mm (H) × 80 mm (W) × 62 mm (L). Thickness of layers: white (enamel) = 1.5 mm, yellow (dentin) = 1.0 mm, and red (pulp) = 2.0 mm.

The test material consisted of multi-layered drilling plates manufactured by Nissin Dental Products, Inc. (Kyoto, Japan), designed for compatibility with dental phantom heads to simulate clinical scenarios (Figure 1) [3]. Each plate featured three distinct layers mimicking enamel (in white), dentin (in yellow), and pulp (in red), with varying hardness levels to replicate natural dental tissues. Drilling was performed using a dental handpiece (model identical across sites) and uniform burs (fissure), with rotational speeds (RPM) controlled as follows: enamel (150,000–200,000 RPM), dentin (100,000–150,000 RPM), and pulp (80,000–100,000 RPM). Procedures were conducted in an ergonomic body posture, without the use of magnifying loupes, to reflect typical preclinical training conditions and ensure consistent visual assessment [24].

### 2.5. Experimental Procedure

At each institution, five participants (T1–T5) independently performed predefined drilling exercises on five samples (S1–S5) [25]: (1) enamel only, (2) enamel and dentin, (3) enamel only (tooth-shaped areas), (4) enamel and dentin (tooth-shaped areas), and (5) all layers (enamel, dentin, and pulp). All participants were instructed to follow the standardized RPM when performing the tasks. Each sample was drilled once per tester, and the test date was recorded for each sample. Exercises were designed to assess the material’s performance across clinically relevant scenarios, with color-coded samples ensuring accurate identification and traceability, made in a randomized order for the different participants.

### 2.6. Evaluation and Data Collection

**Figure A3:**
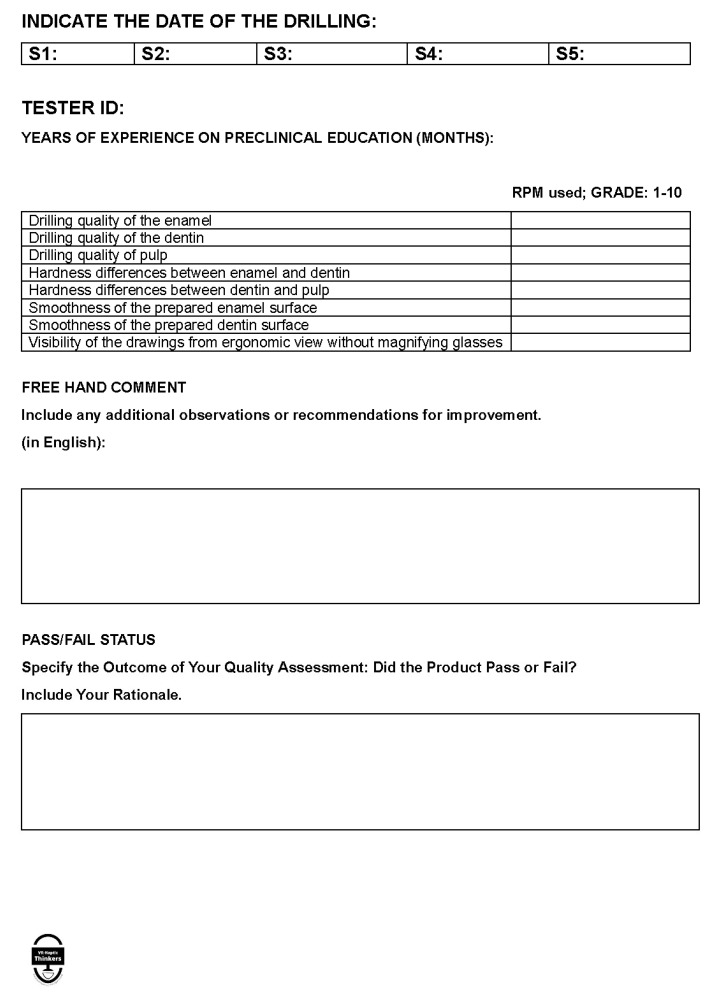
The standardized assessor form used in the study.

Each participant independently assessed their subjective drilling experience using a standardized assessment form (Figure A3) provided in the assessor guidelines. The form included quantitative ratings (1–10 scale, where 1 = poor and 10 = excellent) for the following parameters [26,27,28,29]:

- Drilling quality of enamel;
- Drilling quality of dentin;
- Drilling quality of pulp;
- Hardness differences between enamel and dentin;
- Hardness differences between dentin and pulp;
- Smoothness of prepared dentin surface;
- Visibility of drilling patterns from an ergonomic viewpoint (without magnification).

Qualitative feedback was collected via a free-text section for additional observations or recommendations [30,31]. Each plate received a binary pass/fail grade based on overall performance, with testers providing a rationale for their assessment [32]. The form also recorded testers’ months of preclinical experience and the RPM settings used.

### 2.7. Data Management and Statistical Analysis

Data were collected anonymously to ensure integrity and minimize bias. Quantitative ratings were analyzed using IBM SPSS Statistics (version 29.0.0 or higher, IBM Deutschland GmbH, Böblingen, Germany). Mean scores and standard deviations were calculated for each parameter, and inter-rater reliability was assessed using Krippendorff’s alpha [33]. Linear regression was used to test for potential associations between ratings and educator experience, and the Mann–Whitney *U* test was used to find potential similarities between the pass and fail groups. Qualitative feedback was thematically analyzed to identify common observations and recommendations. All data were stored securely in compliance with the GDPR [34].

## 3. Results

### 3.1. Quantitative Evaluation

Of the 15 dental institutions invited, 14 participated, yielding 70 evaluators with a mean preclinical teaching experience of 112.9 months (approximately 9.4 years; SD = 116.7 months). Table 1 summarizes the quantitative evaluation of the multi-layered drilling plates across all assessed parameters. Mean scores (± standard deviation) for drilling quality were as follows: enamel, 7.80 ± 1.55 (95% CI [7.46, 8.20]); dentin, 7.27 ± 1.94 (95% CI [6.81, 7.73]); and pulp, 7.48 ± 2.33 (95% CI [6.92, 8.04]). The perceived hardness difference between enamel and dentin scored 7.09 ± 2.56 (95% CI [6.47, 7.70]), while the transition between dentin and pulp scored slightly lower at 6.86 ± 2.46 (95% CI [6.26, 7.45]). Surface smoothness post drilling received high mean scores for both enamel (8.17 ± 1.55, 95% CI [7.80, 8.54]) and dentin (8.17 ± 1.57, 95% CI [7.80, 8.55]). The visibility of drilling patterns from an ergonomic perspective (without magnifying loupes) was rated highly at 8.56 ± 1.58 (95% CI [8.17, 8.92]).

**Figure 2:**
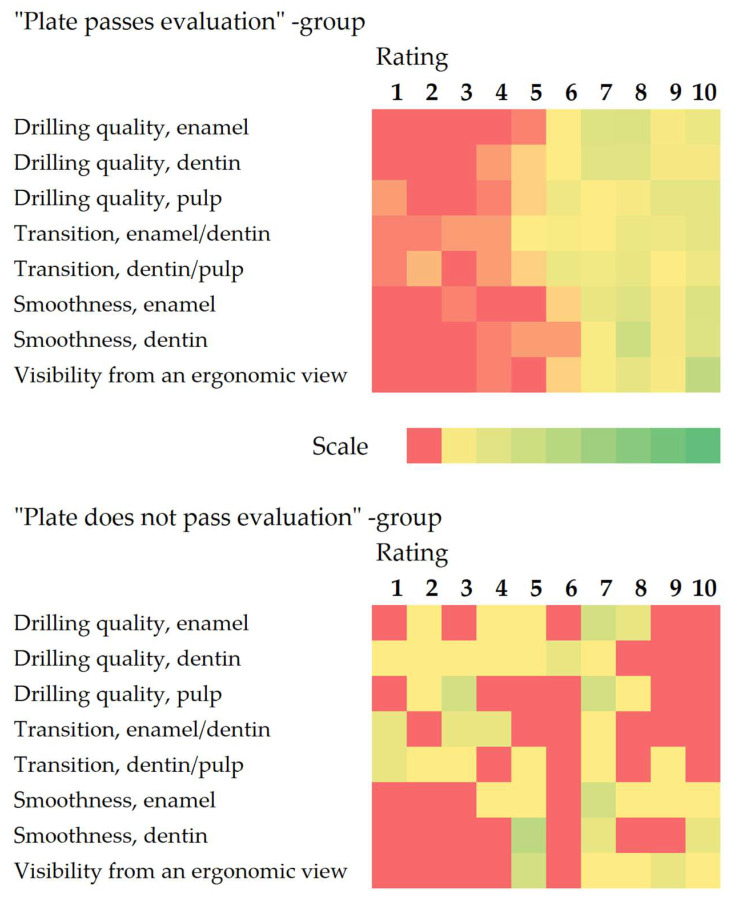
Distribution of ratings. Results shown as heatmaps with the color scales set between zero and the maximum number of replies for the group (“pass” group *n* = 62; “fail” group *n* = 8).

Inter-rater reliability was poor, with Krippendorff’s alpha values of 0.449 for evaluators assigning failing scores and 0.211 for those assigning passing scores, indicating variability in assessments likely due to diverse evaluator backgrounds. Figure 2 illustrates the distribution of ratings for pass and fail groups, showing greater variability in passing scores, particularly for dentin and pulp drilling quality.

Linear regression analysis revealed a significant relationship between teaching experience and assessment ratings (F(8, 57) = 2.412, *p* = 0.026, R^2^ = 0.253, and adjusted R^2^ = 0.148). Specifically, the drilling quality of enamel (*p* = 0.002) and the visibility of drilling patterns (*p* = 0.019) were significantly influenced by evaluators’ experience, suggesting that more experienced educators rated these parameters more favorably.

### 3.2. Qualitative Feedback

Of the 70 participants, 63 (90%) assigned a passing grade to the drilling plates. Statistical analysis showed significant differences in rating distributions between pass and fail groups for all parameters except enamel smoothness (*p* = 0.080). Qualitative feedback highlighted the plates’ strengths, including ease of integration into standard phantom heads, clear visualization of simulated anatomical structures, and straightforward setup. However, 22 participants (31%) noted that the haptic feedback for dentin hardness did not fully replicate natural dental tissue, suggesting an area for refinement to enhance simulation realism. Several evaluators recommended incorporating AI-generated virtual patient models to contextualize drilling exercises, emphasizing the plates’ potential to support preclinical training when paired with advanced digital tools.

## 4. Discussion

The integration of innovative tools, such as multi-layered drilling plates, into dental education might enhance the development of clinical skills in a safe environment [1], particularly for students new to handpiece use. However, challenges such as high initial costs and resistance to change among educators might impede widespread adoption [35,36]. The results from this multi-institutional study, which was one of the first, according to the authors’ best knowledge, involving dental educators with diverse backgrounds, demonstrated the plates’ potential. Ongoing refinement and international collaboration might help to further standardize training tools in dental education. Although VR-haptic simulators are becoming increasingly popular in dental education, physical models remain irreplaceable for preclinical training, as they provide a more direct and tangible experience of resistance and vibration feedback, which are essential to tactile learning and confidence-building in the early training phases [12,37].

The drilling plates received high ratings for surface smoothness and ergonomic visibility, reflecting their suitability for early psychomotor skill development [9]. However, lower scores for tissue transitions indicated challenges in replicating tactile differentiation, a critical skill for restorative dentistry [10,38]. This aligns with findings on 3D-printed models, where intermediate tissues, such as dentin, remained difficult to simulate haptically [7,9,10,11]. The plates’ layered structure and color coding supported tissue identification, but further improvements in haptic realism might be needed.

The high pass rate and positive feedback on ease of use underscored the plates’ value as a training and assessment tool, aligning with competency-based education principles. The wide SDs suggested that participant perceptions varied, potentially influenced by cultural or experiential differences in preclinical training [39]. The relatively high SDs observed in scores, particularly for pulp drilling quality and tissue transitions, likely reflect inter-rater variability. This variability may stem from participants’ diverse teaching experiences and differing institutional practices, which could influence expectations and perceptions of the drilling plates’ haptic and tactile qualities [40]. Such discrepancies highlight the challenge of achieving consistent evaluations across varied expertise levels and suggest a need for standardized rater training or calibration in future studies to enhance inter-rater reliability [41,42]. Nonetheless, the drilling plates might provide additional teaching opportunities, as they could offer a means for structured, objective assessments that are predefined, validated, and reproducible—a growing requirement for dental educators to meet the modern principles of competency-based education [11,43].

However, this study had limitations. The results relied on expert evaluations without student outcome data, limiting insights into long-term learning effects. Given the diverse backgrounds of participants, there might be additional factors that impacted the results, such as variations in familiarity with specific techniques (e.g., orthodontists potentially being less familiar with conservative/endodontic procedures) [44,45], lighting, drill models, handpiece conditions, and ambient noise [46], alongside the likely scenario that each institution might deploy phantom head systems and mounting fixtures from different brands, introducing extra variables. Additionally, the relatively low inter-rater reliability, possibly caused by the wide age range of the participants and differences in their training and professional experience, indicated some level of disagreement among the participants regarding the quality of the drilling plates. This disagreement might have impacted the internal validity of the study by introducing measurement error, despite pre-study calibration efforts, including a video meeting and standardized guidelines. However, as a pilot study, the multi-institutional design and standardized protocols provided preliminary insights into the performance of the novel multi-layered drilling plates.

Still, the drilling plates offered a promising tool for preclinical training, with strengths in usability and assessment potential. Future research should compare the plates with existing models nationally and internationally, especially in terms of cost, durability, and simulation realism [47,48]. Efforts should also be made to include student performance metrics under a more standardized setting to address these gaps. Efforts might also be made to address haptic limitations, standardize equipment compatibility, and explore AI integration [49], which could further enhance their educational impact [50,51], supported by continued multi-institutional collaboration. To address the identified limitations, subsequent studies should incorporate stronger standardization settings, inter-rater reliability training, and statistical adjustments to account for evaluator variability. Future evaluations should include qualitative student feedback on satisfaction and quantitative metrics, such as practice scores and stress levels, to validate the educational impact [52,53]. It would also be important to assess whether there is any variation between different dental specialties. As the global dental community switches its focus from treatment to holistic oral health promotion [54,55], efforts would also be needed from dental educators to integrate approaches with new digital tools such as AI and mHealth into dental curricula [56,57], so that future dentists are equipped with preventive, interdisciplinary, and evidence-based approaches to promote community oral healthcare [58,59].

## 5. Conclusions

The multi-layered drilling plate demonstrated potential as a tool for preclinical dental training, with high educator ratings for drilling quality, smoothness, and visibility, supporting its use for skill development and assessment. However, without direct comparisons to existing training tools, its relative efficacy and realism remain preliminary. Limitations, including reliance on expert evaluations without student outcome data and poor inter-rater reliability due to diverse evaluator backgrounds, suggest the need for cautious interpretation of its effectiveness. Refinements to improve tactile differentiation, particularly for dentin, are needed. Future multi-institutional research should include controlled comparisons with standard tools and student performance metrics to validate the plate’s educational utility and inform its potential integration into dental curricula.

## Figures and Tables

**Table 1:** Dental plate quantitative assessment results. Mean scores with standard deviations (SDs) and 95% confidence intervals (CIs) are shown.

Parameter	*n*	Mean (SD)	95% CI
Drilling quality, enamel	69	7.80 (1.55)	[7.42; 8.17]
Drilling quality, dentin	70	7.27 (1.94)	[6.81; 7.73]
Drilling quality, pulp	69	7.48 (2.33)	[6.92; 8.04]
Transition, enamel/dentin	69	7.09 (2.56)	[6.47; 7.70]
Transition, dentin/pulp	69	6.86 (2.46)	[6.26; 7.45]
Smoothness, enamel	70	8.17 (1.55)	[7.80; 8.54]
Smoothness, dentin	70	8.17 (1.57)	[7.80; 8.55]
Visibility from an ergonomic view	70	8.56 (1.58)	[8.18; 8.93]

*n* = number of submitted answers by participants.

## Data Availability

Data will be made available upon reasonable request to the corresponding author.

## Abbreviations

The following abbreviations are used in this manuscript:

AI	Artificial intelligence
CI	Confidence interval
EU	European Union
RPM	Revolutions per minute
SD	Standard deviation
VR	Virtual reality
